# Changes of blood biochemistry in the rabbit animal model in atherosclerosis research; a time- or stress-effect

**DOI:** 10.1186/1476-511X-10-139

**Published:** 2011-08-14

**Authors:** Ismene A Dontas, Katerina A Marinou, Dimitrios Iliopoulos, Nektaria Tsantila, George Agrogiannis, Apostolos Papalois, Theodore Karatzas

**Affiliations:** 1 Laboratory of Experimental Surgery and Surgical Research "N.S. Christeas", School of Medicine, University of Athens, Greece; 2 Laboratory for Research of the Musculoskeletal System, KAT Hospital, School of Medicine, University of Athens, Greece; 3 Greek Ministry of Rural Development and Food, Department of Diagnosis for Porcine Diseases, Athens, Greece; 4 Laboratory of Pesticides Toxicology, Department of Pesticides Control and Phytopharmacy, Benaki Phytopathological Institute, Athens, Greece; 5 1st Department of Pathology, School of Medicine, University of Athens, Greece; 6 Experimental - Research Center ELPEN Pharma, Pikermi, Greece; 7 2nd Department of Propedeutic Surgery, School of Medicine, University of Athens, Greece

**Keywords:** Animal model, rabbit, atherosclerosis, blood biochemistry, aging, stress, time-related changes, control animals

## Abstract

**Background:**

Rabbits are widely used in biomedical research and especially as animal models in atherosclerosis studies. Blood biochemistry is used to monitor progression of disease, before final evaluation including pathology of arteries and organs. The aim of the present study was to assess the consistency of the biochemical profile of New Zealand White rabbits on standard diet from 3 to 6 months of age, during which they are often used experimentally.

**Methods and results:**

Eight conventional male 3-month-old New Zealand White rabbits were used. Blood samples were taken at baseline, 1, 2 and 3 months later. Plasma glucose, total cholesterol, high-density lipoprotein cholesterol, low-density lipoprotein cholesterol, triacylglycerol concentrations, and alanine aminotransferase, aspartate aminotransferase, alkaline phosphatase, gamma glutamyl transferase activities and malondialdehyde were measured. Statistically significant time-related changes were observed in glucose, total cholesterol and triacylglycerol, which were not correlated with aortic lesions at 6 months of age. Similarly, hepatic enzyme activity had significant time-related changes, without a corresponding liver pathology.

**Conclusions:**

Age progression and stress due to single housing may be the underlying reasons for these biochemistry changes. These early changes, indicative of metabolic alterations, should be taken into account even in short-term lipid/atherosclerosis studies, where age and standard diet are not expected to have an effect on the control group of a study.

## Background

Rabbits are widely used in biomedical research and especially as animal models in atherosclerosis studies [1,2]. For the induction of the non-genetic hyperlipidemic rabbit animal model of atherosclerosis, several atherogenic diets of varying cholesterol concentrations and administration times are applied [3-5]. Blood biochemistry is used to monitor progression of disease, before final evaluation including pathology of arteries and organs [1,6,7]. With blood biochemistry monitoring, the number of animals used is reduced as they serve as their own controls, and additionally, minimal discomfort is induced by blood sampling.

We considered that time- or age-related changes of blood biochemistry of control animals fed standard diets should be thoroughly investigated, as, in most studies, their values are compared to those of the experimental groups [1,8,9]. Blood biochemical parameters have been shown to be subject to change with increasing age in many animal species [10-12]. The aim of the present study therefore was to assess the consistency of the biochemical profile of New Zealand White (NZW) rabbits on a standard rabbit diet from 3 to 6 months of age, which is an age during which they are often used experimentally [3,9].

## Results

**Figure 1:**
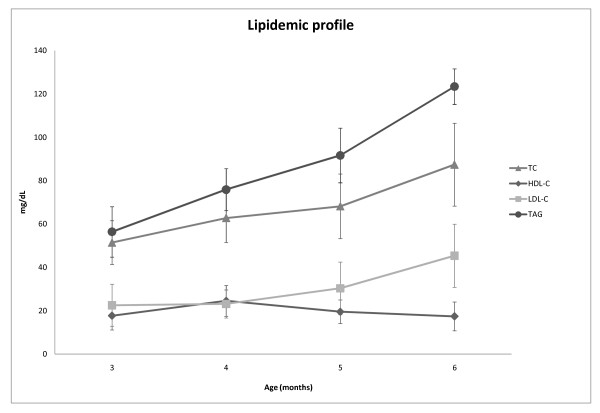
**Lipidemic profile**. Changes of lipidemic profile of New Zealand White rabbits on normal rabbit diet at the age of 3, 4, 5 and 6 months. Values are means of 8 rabbits (mg/dL), bars indicate SD. TC: total cholesterol, HDL-C: high-density lipoprotein cholesterol, LDL-C: low-density lipoprotein cholesterol, TAG: triacylglycerol.

**Figure 2:**
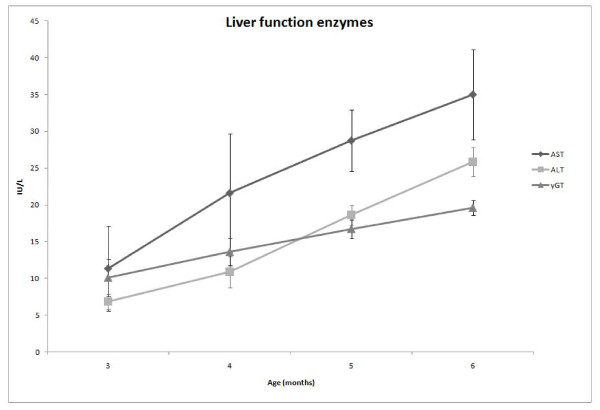
**Liver function enzymes**. Changes of liver function enzymes of New Zealand White rabbits on normal rabbit diet at the age of 3, 4, 5 and 6 months. Values are means of 8 rabbits (IU/L), bars indicate SD. AST: aspartate aminotransferase, ALT: alanine aminotransferase, γGT: gamma glutamyl transferase.

The results of all measured parameters are presented in Table 1 (statistical difference is indicated in both levels of p < 0.05 and p < 0.001). Results are also depicted as graphs in Figures 1 and 2.

**Table 1:** Plasma parameters and body weight of NZW rabbits measured during the observation period

Parameter (units)	Baseline	1^st ^month	2^nd ^month	3^rd ^month
Glucose (mg/dL)	134.62 (8.07)	153.50 (7.76)†	169.50 (8.66)†	177.50 (7.60)†

TC (mg/dL)	51.38 (10.10)	62.75 (11.30)†	68.13 (14.90)*	87.38 (19.15)†

HDL-C (mg/dL)	17.63 (6.52)	24.50 (7.13)*	19.50 (5.48)	17.38 (6.67)

LDL-C (mg/dL)	22.48 (9.68)	23.08 (6.49)	30.30 (12.11)	45.33 (14.55)†

TAG (mg/dL)	56.37 (11.62)	75.87 (9.67)†	91.62 (12.6)†	123.38 (8.19)†

ALT (IU/L)	6.87 (1.00)	10.87 (2.10)†	18.62 (1.30)†	25.87 (1.95)†

AST (IU/L)	11.37 (5.75)	21.62 (8.07)*	28.75 (4.16)†	35.00 (6.11)†

ALP (IU/L)	40.12 (4.35)	74.75 (15.09)†	91.87 (12.82)†	168.25 (19.88)†

γGT (IU/L)	10.12 (2.47)	13.62 (1.84)†	16.75 (1.28)†	19.62 (1.00)†

MDA (nmol/L)	1.16 (0.20)	1.26 (0.16)	1.59 (0.10)*	2.08 (0.19)†

Body weight (kg)	3.05 (0.26)	3.42 (0.18)†	3.46 (0.26)†	3.49 (0.21)†

Baseline sampling corresponds to the age of 3 months, 1^st ^month sampling to the age of 4 months, 2^nd ^month sampling to the age of 5 months and 3^rd ^month sampling to the age of 6 months. Values are means of 8 rabbits (SD). Statistical significance is indicated with *p < 0.05 vs baseline and † p < 0.001 vs baseline.

All values are presented as Mean (SD)

See Results section for abbreviations

*p < 0.05 vs baseline; † p < 0.001 vs baseline

### Biochemical values

Glucose values increased significantly during the study in all three months (p < 0.001) when compared with baseline values (Table 1, Figure 1).

Total cholesterol (TC) values increased significantly during the study in all three months when compared with baseline values; however, the TC increase in the 1st and 3rd month (p < 0.001) was greater than the one in the 2nd month (p < 0.05) (Table 1, Figure 1).

High-density lipoprotein cholesterol (HDL-C) values increased significantly during the study only in the 1st month (p < 0.05) when compared with baseline values (Table 1, Figure 1).

Low-density lipoprotein cholesterol (LDL-C) values increased significantly during the study only in the 3rd month (p < 0.001) when compared with baseline values (Table 1, Figure 1).

Triacylglycerol (TAG) values increased significantly during the study in all three months (p < 0.001) when compared with baseline values (Table 1 Figure 1).

### Liver function

Alanine aminotransferase (ALT) activity increased significantly during the study in all three months (p < 0.001) when compared with baseline values (Table 1, Figure 2).

Aspartate aminotransferase (AST) activity increased significantly during the study in all three months when compared with baseline values. However, the AST activity increase in the 2nd and 3rd month (p < 0.001) was greater than the one in the 1st month (p < 0.05) (Table 1, Figure 2).

Alkaline phosphatase (ALP) activity increased significantly during the study in all three months (p < 0.001) when compared with baseline values (Table 1).

Gamma glutamyl transferase (γGT) activity increased significantly during the study in all three months (p < 0.001) when compared with baseline values (Table 1, Figure 2).

### Antioxidant evaluation

Malondialdehyde (MDA) values significantly increased only in the 2nd and 3rd month of the study. Moreover, the increase in the 3rd month was greater than the one in the 2nd month (p < 0.001 and p < 0.05, respectively) (Table 1).

### Body weight

Rabbit body weight increased significantly over time. This increase is observed in all three months (p < 0.001) when compared with the respective baseline values (Table 1).

### Pathology

The control rabbits did not have any atherosclerotic lesions in their aorta, as expected from other similar studies. More specifically, the aortic intima had a normal thickness, there was no foam cell accumulation or mononuclear infiltrates, and no lipid core or fibrous cap formation.

No liver pathology was observed at necropsy, including fatty infiltration.

## Discussion

Various studies have reported differences in laboratory animal blood biochemistry parameters, as well as haematological parameters, related to species, strain, sex and age [11,13-15]. Blood collection procedures related to duration of preceding fasting, time of sampling, time of samples to stand, hemolysis, use of plasma instead of serum, storage until time of measurement, method of analysis, are factors that can affect blood parameters measured [11,13]. Although there is a multitude of reports on the lipidemic changes of heritable hyperlipidemic rabbits in chronic studies [16-19], there are relatively fewer reports on detailed changes in normal rabbits that are used as comparative controls [20,21].

In the present study we sought to verify if during a short-term (3-month) study, normal young male NZW rabbits under a normal diet, which are often used as controls, have a stable biochemical profile, by examining their plasma values after a 12-hour fasting period. Particular attention was paid to the blood sampling procedures in order to exclude preanalytical variation. With the use of mild short-acting sedation, stress related to the blood-sampling procedure was avoided. Additionally, auricular vasodilation is prominent under sedation, which makes the sampling procedure simple and brief. The person in charge of the sampling had years of experience and training. The previously mentioned 12/12h lighting schedule of the animal house has been shown to elicit the fewest variations in blood biochemistry parameters [22] and the same sampling time was followed in order to avoid potential diurnal variations [11].

The baseline blood biochemical values of our study at 3 months were similar to those of other researchers in NZW rabbits [13,23,24]. In our study, plasma glucose levels of male NZW rabbits increased from the age of 3 months (135 mg/dL) throughout 6 months (177 mg/dL), even from the 4^th ^month of age, with a statistically significant difference (p < 0.001 vs baseline, Table 1). Glucose appears to vary in studies with male NZW rabbits, with normal values at 112 mg/dL [14] or 187 mg/dL (10.42 mmol/L) [25]. As glucose values are reported to present diurnal variation and affected when animals are frightened when handled or restrained without anesthesia [11], different values between studies may be due these reasons. In the present study, as previously mentioned, all samplings were carried out under sedation and at the same time of the morning.

Total cholesterol values similarly increased statistically significantly throughout the study, beginning at 51 and reaching 87 mg/dL (p < 0.001 vs baseline, Table 1, Figure 1). In contrast to our findings, Orlandi et al. found minimal differences in total cholesterol values of 4-month-old and 5-year-old NZW rabbits, which ranged between 28 and 34 mg/dL [21]. Another study on younger (2-month-old) NZW rabbits reported 81 mg/dL (2.11 mmol/L) [24].

HDL-C values presented a small increase one month after our study start (p < 0.05 vs baseline), similar to other studies (23.5 mg/dL) [24] returning to baseline values at the end. LDL-C values however significantly increased at 6 months of age (p < 0.001 vs baseline). Triacylglycerol initial values were 56 mg/dL and reached 123 mg/dL at 6 months of age (p < 0.001 vs baseline).

Additionally, the activity of hepatic enzymes ALT and AST increased with age In the present study. This is in agreement with Matsuzawa et al., who studied age-related biochemical changes in a large number of other animals (monkeys, dogs and rats) [11]. Their study supported that as a general trend, these hepatic enzymes increase with age, which however does not necessarily reflect a corresponding liver pathology, as their increase has been noted to occur due to fear of venepuncture. The authors also supported that liver enzymes are not elevated in heparinized blood. Therefore, the significant increase of values in the samples of the present study was not due to an anticoagulant effect, but most probably was age-related.

MDA also increased significantly towards the end of the study (p ≤ 0.001 vs baseline, Table 1). MDA has been the most studied product of polyunsaturated fatty acid peroxidation, indicating oxidative stress [26].

The aortas of the rabbits of the present study had normal both macro- and microscopical appearance, which is not compatible with the observed biochemical changes. Other studies with control groups of rabbits of 6 months of age had no pathologic findings in their aortas similarly to ours [3,9].

As previously mentioned, the rabbits of this study consisted the control group of an experimental atherosclerosis study. It is acknowledged that their number (n = 8) may be considered small compared to other studies that have as main objective to establish reference values of blood parameters. However, their values' SD did not have a wide range and it is also ethically desirable to use as few animals as possible in experimental research [27]. Similar numbers of rabbits per group to ours have also been used in other experimental atherosclerosis studies [1,9,21].

Diet composition is known to influence blood biochemical parameters. A change in lipid blood parameters in a short period of time could be due to a change of diet lipid composition. However, the rabbits of the present study were fed the same diet (2.5% fat content) in the breeding establishment and subsequently in our experimental establishment. Therefore, their lipid profile change cannot be attributed to a change of diet.

New Zealand White rabbits are the breed that has been and continues to be used in many studies of atherosclerosis research [1,28,29]. However, it has not been sufficiently taken into account that this breed has been also considered a spontaneous model of diabetes [30]. It could be possible that with *ad libitum *feeding throughout time, diabetes could emerge. This is supported by the finding that plasma glucose levels were significantly increased compared to baseline already by the 4^th ^month of age, and continued to rise. Additionally, this could be the beginning of a "metabolic syndrome", which perhaps did not have the time to manifest itself fully, because the animals had to be euthanized at 6 months of age. It is possible that with time, their metabolism undergoes an age-related change. It consists a limitation of the present study that it was not considered to monitor their blood pressure non-invasively throughout this period, as this would assist in accepting or rejecting the metabolic syndrome hypothesis. A longer observation period in a future study might also give answers. Aging has been proved to induce blood biochemistry changes in many animal species [10-12].

A possibility that the prenatal environment could have had a role in these biochemical alterations was also considered. The breeding establishment may have had some adverse effect on the pregnant does during our rabbits' prenatal life, which is practically impossible to investigate retrospectively. It is well known that maternal stress and nutritional imbalance during pregnancy affects fetal postnatal development adversely and can lead to several diseases in adult life, such as metabolic diseases, hypertension, renal insufficiency, etc. [31,32], which consists the "fetal origins" hypothesis [33]. This possibility cannot be ruled out.

Another factor that may have developed a stressful situation to our rabbits is their housing conditions. In the breeding establishment, they were housed in rows of cages in physical contact with each other. In their new housing condition in the experimental establishment, they were housed singly in stainless steel cages, with no visual or physical contact. It is well known that isolation stress is a potent stressor [34,35], which very likely induced the biochemical changes. On the contrary, one blood sampling per month by experienced personnel and under the aforementioned precautions cannot be considered a stressing situation. Taking into account their overall experimental housing conditions, the isolated laboratory life of the rabbits appears to be a probable cause for these biochemical changes.

## Conclusions

Normal growth and standard diet in NZW rabbits induced statistically significant time-related changes in glucose and lipid profile from 3 to 6 months of age, which were not correlated with aortic lesions at 6 months. Similarly, hepatic enzyme activity had significant time-related changes, without a corresponding liver pathology.

Age progression and stress due to single housing may be the underlying reasons for these changes. These early changes in the rabbit animal model, indicative of metabolic alterations, should be taken into account even in short-term protocols of lipid/atherosclerosis studies, where age and standard diet are not expected to have an effect on control animals.

## Methods

### Laboratory animals

Eight conventional male NZW rabbits, with a body weight of 3.05 ± 0.26 Kg (mean ± SD), at the age of three months old, purchased from a Greek approved commercial breeder, consisted the control group of an experimental atherosclerosis study. The local Veterinary Authorities of the Athens Prefecture evaluated and approved (License No. K/950) the study, according to the Greek regulations that have been harmonized to the European Directive 86/609/EEC. The rabbits were kept singly in stainless steel cages with free access to food and tap water for a period of three months. The animal house conditions consisted of 20 ± 2°C and 60 ± 5% relative humidity, under a 12/12h light/dark cycle. The animals were handled according to standards imposed by the European Directive 86/609/EEC. The animals received standard rabbit balanced diet (chemical composition: total fatty acids 2.5%, cellulose 18.5%, total protein 16.5%, water 13%, ash 11%, calcium 1.4%, lysine 0.6%, methionine-cystine 0.55%, phosphorus 0.55%, sodium 0.25%).

### Blood samplings and biochemical values

In the context of the atherosclerosis study, all rabbits were subjected to monthly blood samplings. They were fasted 12 hours prior to blood sampling. They were mildly sedated (ketamine hydrochloride 12 mg/kg, xylazine 2.5 mg/kg body weight, im) for the procedure, in order to avoid stress impact. Blood samples withdrawn from the auricular artery of animals were placed into Wassermann tubes containing anticoagulant at 0, 1, 2 and 3 months of the experimental procedure. The 1^st ^sampling was conducted after a 10-day acclimatization period. Diurnal variations were avoided by sampling the animals during the same time of the day (09:30 - 11:00). Plasma was separated by centrifugation at 3500 rpm for 15 min. Plasma total cholesterol (TC), high-density lipoprotein cholesterol (HDL-C), low-density lipoprotein cholesterol (LDL-C), triacylglycerol (TAG) concentrations, alanine aminotransferase (ALT), aspartate aminotransferase (AST), alkaline phosphatase (ALP) and gamma glutamyl transferase (γGT) activities were measured by commercial enzymatic test kits according to the manufacturer's instructions (Biomerieux, Lyon, France) using an automatic analyser (Type 7170A, Hitachi, Tokyo, Japan). Malondialdehyde (MDA) was calculated by the thiobarbituric acid reactive substances manual method as described by Yagi [36]. At the end of the experimental study and after the last blood sampling under sedation, the rabbits were euthanized with sodium thiopental (30 mg/kg iv) for the removal and examination of the aorta and liver.

### Tissue samples

The aorta was removed from the aortic arch to the iliac bifurcation and cut longitudinally along the mid-ventral wall. The aorta was then fixed flatly in 10% phosphate buffered formalin solution. The luminal surface of each aortic specimen was photographed and the image was stored electronically. Sections from all specimens were obtained from three standard sites (immediately distal to the branch of the left subclavian artery, at the seventh intercostal artery and immediately posterior to the celiac artery). These samples were embedded in paraffin blocks and stained with hematoxylin-eosin. In brief, parameters evaluated were: intimal thickening, foam cell accumulation, mononuclear infiltrates lipid core and fibrous cap formation.

The liver was removed en bloc. Standard sections were taken, embedded in paraffin blocks for hematoxylin-eosin and were examined for alterations of architecture, fatty infiltration and fibrosis.

### Statistical analysis

Data was expressed as mean values ± standard deviation (SD). The Kolmogorov-Smirnov test was utilized for normality analysis of the parameters. One-way analysis of variance (ANOVA) was used.

All tests were two-sided, statistical significance was set at p < 0.05. All analyses were carried out using the statistical package SPSS vr 16.00 (Statistical Package for the Social Sciences, SPSS Inc., Chicago, Ill., USA).

## Abbreviations

NZW: New Zealand White; TC: total cholesterol; HDL-C: high-density lipoprotein cholesterol; LDL-C: low density lipoprotein cholesterol; TAG: triacylglycerol; MDA: malondialdehyde; ALT: alanine aminotransferase; AST: aspartate aminotranferase; γ-GT: gamma glutamyl transferase; SD: standard deviation; SPSS: Statistical Package for the Social Sciences.

## Competing interests

The authors declare that they have no competing interests.

## Authors' contributions

ID conceived the study design, coordinated the experiments, participated in the blood samplings, euthanasias, and wrote the manuscript. KM was responsible for the experimental study, including general overview of the animals, participated in the blood samplings, euthanasias and contributed to the preparation of the manuscript. DI and TK executed the removal of tissues. NT assisted in the preparation of the manuscript text, table and figures. GA performed and evaluated the tissue samples' pathology. AP contributed to the study design and experiments. TK contributed to the preparation of the manuscript, and the discussion and interpretation of the findings. All authors read and approved the final manuscript.

## References

[B1] Aguilera CM Ramirez-Tortosa MC Mesa MD Ramirez-Tortosa CL Gil A Sunflower, virgin-olive and fish oils differentially affect the progression of aortic lesions in rabbits with experimental atherosclerosis Atherosclerosis 2002 162 335 344 10.1016/S0021-9150(01)00737-7 11996953

[B2] Yanni AE The laboratory rabbit: an animal model of atherosclerosis research Lab Anim 2004 38 246 256 10.1258/002367704323133628 15207035

[B3] Hatipoglu A Kanbagli O Balkan J Kucuk M Cevikbas U Aykac-Toker G Berkkan H Uysal M Hazelnut oil administration reduces aortic cholesterol accumulation and lipid peroxides in the plasma, liver, and aorta of rabbits fed a high- cholesterol diet Biosci Biotechnol Biochem 2004 68 2050 57 10.1271/bbb.68.2050 15502349

[B4] Gonzalez-Santiago M Martin-Bautista E Carrero JJ Fonolla J Baro L Bartolome MV Gil-Loyzaga P Lopez-Huertas E One-month administration of hydroxytyrosol, a phenolic antioxidant present in olive oil to hyperlipemic rabbits improves blood lipid profile, antioxidant status and reduces atherosclerosis development Atherosclerosis 2006 188 35 42 10.1016/j.atherosclerosis.2005.10.022 16300770

[B5] Ren M Rajendran R Ning P Huat BTK Nam OC Watt F Jenner A Halliwell B Zinc supplementation decreases the development of atherosclerosis in rabbits Free Radical Biol Med 2006 41 222 25 10.1016/j.freeradbiomed.2006.03.017 16814102

[B6] Marinou KA Georgopoulou K Agrogiannis G Karatzas T Iliopoulos D Papalois A Chatziioannou A Magiatis P Halabalaki M Tsantila N Skaltsounis LA Patsouris E Dontas IA Differential effect of Pistacia vera extracts on experimental atherosclerosis in the rabbit animal model: an experimental study Lipids Health Dis 2010 9 73 10.1186/1476-511X-9-73 20633299 PMC2917426

[B7] Tsantila N Karantonis HC Perrea DN Theocharis SE Iliopoulos DG Iatrou C Antonopoulou S Demopoulos CA Atherosclerosis regression study in rabbits upon olive pomace polar lipid extract administration Nutr Metab Cardiovasc Dis 2010 20 740 7 10.1016/j.numecd.2009.06.008 19748252

[B8] Hakimoglu F Kızıl G Kanay Z Kızıl M Isı H The effect of ethanol extract of Hypericum lysimachioides on lipid profile in hypercholesterolemic rabbits and its in vitro antioxidant activity Atherosclerosis 2007 192 113 122 10.1016/j.atherosclerosis.2006.07.013 16901489

[B9] Jenner A Ren M Rajendran R Ning P Huat BT Watt F Halliwell B Zinc supplementation inhibits lipid peroxidation and the development of atherosclerosis in rabbits fed a high cholesterol diet Free Radic Biol Med 2007 42 559 66 10.1016/j.freeradbiomed.2006.11.024 17275688

[B10] Ihrig M Tassinary LG Bernacky B Keeling ME Hematologic and seum biochemical reference intervals for the chimpanzee (Pan troglodytes) categorized by age and sex Comp Med 2001 51 30 7 11926299

[B11] Matsuzawa T Nomura M Unno T Clinical pathology reference ranges of laboratory animals J Vet Med Sci 1993 55 351 62 10.1292/jvms.55.351 8357905

[B12] Mohri M Sharifi K Eidi S Hematology and serum biochemistry of Holstein dairy calves: age related changes and comparison with blood composition in adults Res Vet Sci 2007 83 30 39 10.1016/j.rvsc.2006.10.017 17188315

[B13] Wolford ST Schroer RA Gohs FX Gallo PP Brodeck M Falk HB Ruhren R Reference range data base for serum chemistry and hematology values in laboratory animals J Toxicol Environ Health 1986 18 161 188 10.1080/15287398609530859 3712484

[B14] Jeklova E Leva L Knotigova P Faldyna M Age-related changes in selected haematology parameters in rabbits Res Vet Sci 2009 86 525 528 10.1016/j.rvsc.2008.10.007 19041105

[B15] Olayemi FO Nottidge HO Effect of age on the blood profiles of the New Zealand White rabbit in Nigeria Afr J Biomed Res 2007 10 73 76

[B16] Lind BM Littbarski R Hohlbach G Moller KO Long-term investigations of serum cholesterol, serum triglyceride, and HDL cholesterol in heritable hyperlipidemic rabbits Zeitschrift fur Versuchstierkunde 1990 33 245 9 2082621

[B17] Mortensen A Frandsen H Reproductive performance and changes in blood lipids in breedig females and in growing Watanabe Heritable Hyperlipidaemic and New Zealand White rabbits Lab Anim 1996 30 252 259 10.1258/002367796780684854 8843050

[B18] Yamada S Ito T Tamura T Shiomi M Age-related changes in serum/plasma biochemical parameters of WHHLMI rabbits Exp Anim 2004 53 159 163 10.1538/expanim.53.159 15153680

[B19] Ying Z Kherada N Kampfrath T Mihai G Simonetti O Desikan R Selvendiran K Sun Q Ziouzenkova O Parthasarathy S Rajagopalan S A modified sesamol derivative inhibits progression of atherosclerosis Arterioscler Thromb Vasc Biol 2011 31 536 42 10.1161/ATVBAHA.110.219287 21183734 PMC5343762

[B20] Spagnoli LG Orlandi A Mauriello A Santeusanio G De Angelis C Lucreziotti R Ramacci MT Aging and atherosclerosis in the rabbit 1: Distribution, prevalence and morphology of atherosclerotic lesions Atherosclerosis 1991 89 11 24 10.1016/0021-9150(91)90003-L 1772469

[B21] Orlandi A Marcellini M Spagnoli LG Aging influences development and progression of early aortic atherosclerotic lesions in cholesterol-fed rabbits Arterioscler Thromb Vasc Biol 2000 20 1123 1136 10.1161/01.ATV.20.4.1123 10764683

[B22] Illera JC Silvan G Lorenzo P Portela A Illera MJ Illera M Photoperiod variations of various blood biochemistry constants in the rabbit Rev Esp Fisiol 1992 48 7 12 1410771

[B23] Abdelhalim MAK Alhadlaq HA Effects of cholesterol feeding periods on blood haematology and biochemistry of rabbits Int J Biol Chem 2008 2 49 53 10.3923/ijbc.2008.49.53

[B24] De La Cruz JP Villalobos MA Carmona JA Martin-romero M Smith-Agreda JM De la Cuesta FS Antithrombotic potential of olive oil administration in rabbits with elevated cholesterol Thrombosis Res 2000 100 305 315 10.1016/S0049-3848(00)00321-2 11113274

[B25] Aleman CL Noa M Mas R Rodeiro I Mesa R Menendez R Gamez R Hernandez C Reference data for the principal physiological indicators in three species of laboratory animals Lab Anim 2000 34 379 385 10.1258/002367700780387741 11072858

[B26] Lin WY Chen CS Wu SB Lin YP Levin RM Wei YH Oxidative stress biomarkers in urine and plasma of rabbits with partial bladder outlet obstruction BJU Int 2010 in press 10.1111/j.1464-410X.2010.09597.x 20875092

[B27] Festing M Howard, Nevalainen, Peretta Reduction by careful design and statistical analysis The COST manual of laboratory animal and use - refinement, reduction, and research 2011 Boca Raton: CRC Press, Taylor & Francis Group 131 149

[B28] Houssen ME Haron MM Metwally SS Ibrahim TM Effects of immunomodulatory drugs on plasma inflammatory markers in a rabbit model of atherosclerosis J Physiol Biochem 2011 67 115 20 10.1007/s13105-010-0055-1 20960084

[B29] Nakazawa G Nakano M Otsuka F Wilcox JN Melder R Pruitt S Kolodgie FD Virmani R Evaluation of polymer-based comparator drug-eluting stents using a rabbit model of iliac artery atherosclerosis Circ Cardiovasc Interv 2011 4 38 46 10.1161/CIRCINTERVENTIONS.110.957654 21205943

[B30] Rees DA Alcolado JC Animal models of diabetes mellitus Diab Med 2005 22 359 370 10.1111/j.1464-5491.2005.01499.x 15787657

[B31] Osmond C Barker DJ Fetal, infant, and childhood growth are predictors of coronary heart disease, diabetes, and hypertension in adult men and women Environ Health Perspect 2000 108 Suppl 3 545 53 10852853 10.1289/ehp.00108s3545 PMC1637808

[B32] Ozanne SE Hales CN Poor fetal growth followed by rapid postnatal catch-up growth leads to premature death Mech Ageing Dev 2005 126 852 854 10.1016/j.mad.2005.03.005 15992609

[B33] Godfrey KM Barker DJP Fetal nutrition and adult disease Am J Clin Nutr 2000 71 Suppl 1344S 1352S 10799412 10.1093/ajcn/71.5.1344s

[B34] Serra M Pisu MG Floris I Floris S Cannas E Mossa A Trapani G Latrofa A Purdy RH Biggio G Social isolation increases the response of peripheral benzodiazepine receptors in the rat Neurochem Int 2004 45 141 8 10.1016/j.neuint.2003.11.013 15082231

[B35] Weiss IC Pryce CR Jongen-Rêlo AL Nanz-Bahr NI Feldon J Effect of social isolation on stress-related behavioural and neuroendocrine state in the rat Behav Brain Res 2004 152 279 95 10.1016/j.bbr.2003.10.015 15196796

[B36] Yagi K Simple assay for the level of total lipid peroxides in blood plasma Meth Mol Biol 1998 108 101 6 10.1385/0-89603-472-0:101 9921519

